# Prognostic value of activity patterns and stress measures for persistent pain and disability in acute neck pain: a 3-month follow-up study

**DOI:** 10.3389/fpain.2025.1686389

**Published:** 2025-11-13

**Authors:** Rita Morf, Cyrill Kernwein, Julia Jaeger, Leah Reicherzer, Jürgen Degenfellner, Sabina Hotz-Boendermaker

**Affiliations:** 1 Zurich University of Applied Sciences (ZHAW), School of Health Sciences, Institute of Physiotherapy, Winterthur, Switzerland; 2 Physiotherapy Medbase Winterthur, Winterthur, Switzerland

**Keywords:** stress, activity patterns, pain, disability, neck pain

## Abstract

**Background:**

Neck pain (NP) represents a significant global health challenge, with a considerable proportion of individuals enduring persistent NP, which is associated with psychological stress. However, it remains unclear whether stress acts as a prognostic factor or emerges as a consequence of ongoing pain. An individual's behavioral response to pain, known as activity patterns (eustress persistence, distress persistence, activity pacing, and fear avoidance), reflects how individuals engage in daily activities and may influence both the experience and course of pain. These patterns have been linked to stress, potentially exacerbating pain intensity and disability. This study aims to investigate the prognostic value of activity patterns, subjective and objective stress in acute NP after three months. Furthermore, it examines the relationship between subjective and objective stress measures.

**Methods:**

This study included participants (*n* = 125) with acute neck pain (NP) (<4 weeks). Baseline stress levels were measured objectively using hair cortisol concentration and subjectively using the Stress and Coping Inventory (SCI). Activity patterns were identified using the Avoidance-Endurance Fast Screen (AE-FS). Linear mixed models (LMM) were used to assess whether stress and activity patterns during the acute phase were prognostic factors for pain and disability three months later. A Pearson correlation was calculated between the subjective and objective stress measures.

**Results:**

Weak correlations were found between subjective and objective stress measures. In the LMM, higher pain intensity during the acute NP phase was associated with increased pain intensity at 3-month follow-up. In terms of disability, both initial pain intensity and “stress due to uncertainty” were associated with higher disability after three months.

**Discussion:**

Only a few consistent prognostic factors for persistent pain and disability have been identified, raising the question of whether current measures capture the most relevant aspects.

**Clinical Trial Registration:** https://www.clinicaltrials.gov published 07/22, identifiers NCT05468684.

## Introduction

Neck pain (NP) affects a large proportion of the global population, with a lifetime prevalence of up to 71% in the general population worldwide (1). Although pain and disability often diminish rapidly around six weeks after pain onset without intervention, both outcomes tend to remain elevated over time (2). The likelihood of symptom recurrence within five years ranges from 50%–85% among individuals with NP, indicating that persistent or recurrent NP is common in this population (3). Identifying and addressing prognostic factors in an acute episode may help alleviate persistent pain. In addition to behavioral influences such as sleep quality (4), psychosocial variables, including emotional health and coping strategies, have been linked consistently with the onset, progression, and recurrence of neck pain, while general physical activity showed limited predictive value (5). Moreover, depression and anxiety have been identified as prognostic factors for a worse outcome at three months in acute NP (6). Stress is another psychological factor that refers to the physical and mental strain individuals experience when facing challenging situations, as the body tries to restore balance (7). For persistent neck pain, a meta-analysis has shown a relationship with psychological stress (8); comparable studies are lacking for acute NP. However, it has been confirmed as a prognostic factor for the persistence of acute low back pain (9). The measurement of stress can be conducted subjectively using questionnaires or objectively through the measurement of hair cortisol levels (10). Evidence for a strong association between objective stress measurement and subjective stress measures remains inconclusive (11–13).

Additional modifiable factors contributing to the development or maintenance of persistent pain include pain-related activity patterns. They reflect a relationship between physical and psychosocial factors, as well as beliefs, and involve behaviors such as fear avoidance, activity pacing, or persistence (14). For instance, persistence entails continuing a task despite the pain, even to the point of severe pain aggravation (15). Persistence can be further categorized into two subtypes: eustress persistence, characterized by focused cognitive distraction and positive mood (15), and distress persistence, characterized by thought suppression, distress, and perseverative behavior (16). Activity pacing represents a strategy that balances activity and regeneration to achieve favorable outcomes (17). Finally, fear avoidance decreases pain-associated activities and involves pain catastrophizing (18). Behavioral research has extensively examined fear avoidance as a risk factor for persistent pain (19–24). Recent findings in participants with acute low back pain showed that baseline persistence and not fear avoidance was associated with persistent pain (25).

In individuals with acute NP, the association of these activity patterns remains understudied to date. Recently, we have suggested that individuals with acute NP who were classified with distress persistence have the highest stress levels compared to the other activity patterns (26). The relationship between activity patterns and stress presents an intriguing area of research. How individuals perceive and regulate stress may be influenced by their activity patterns, which in turn affect how stress impacts the persistence of pain.

Indeed, in individuals with acute low back pain, measurements of saliva cortisol levels taken after awakening, which reflect momentary stress, showed that persistent behavior was associated with lower cortisol levels than other activity patterns (27). Individuals experiencing persistent pain showed markedly higher hair cortisol levels compared to healthy adults (28). However, in our recent findings individuals with acute NP had hair cortisol levels that were not elevated (26). As longitudinal studies are lacking, it is unclear whether hair cortisol concentration, perceived stress levels, and activity patterns can be used to prognose the persistence of NP.

The primary aim of this study was to investigate whether activity patterns and subjective and objective stress measurements, in the acute phase of NP, could serve as prognostic factors for the persistence of pain and disability. Furthermore, the study explored the correlation between subjective and objective assessments. Understanding this interaction could provide valuable insights for tailoring pain management interventions.

## Methods/design

### Study design

This study is part of a larger cohort study investigating clinical, somatic, and psychosocial factors in individuals with acute NP over one year. In this publication, time point 1 (T1), which occurs within four weeks of pain onset, and time point 2 (T2), three months after pain onset, were analyzed. This research project adhered to the STROBE guidelines (29) and was conducted in accordance with the principles outlined in the Declaration of Helsinki. Approval was obtained from the local Ethics Committee (BASEC-No. 2022-00846). More information about the study procedures can be found in the published study protocol (30).

### Participants

The cohort included 125 participants with acute NP (pain onset less than four weeks), who had been pain-free for the previous three months, aged 18–65 years, and proficient in German. Exclusion criteria were recent pregnancy or childbirth, peripheral or central neurological, oncological, chronic pulmonary, or acute psychiatric conditions. Frequent headaches (≥2x monthly) and migraines (≥1x monthly) were excluded due to their high association with NP and moderate evidence of cervical musculoskeletal impairments (31, 32).

### Recruitment

**Figure 1:**
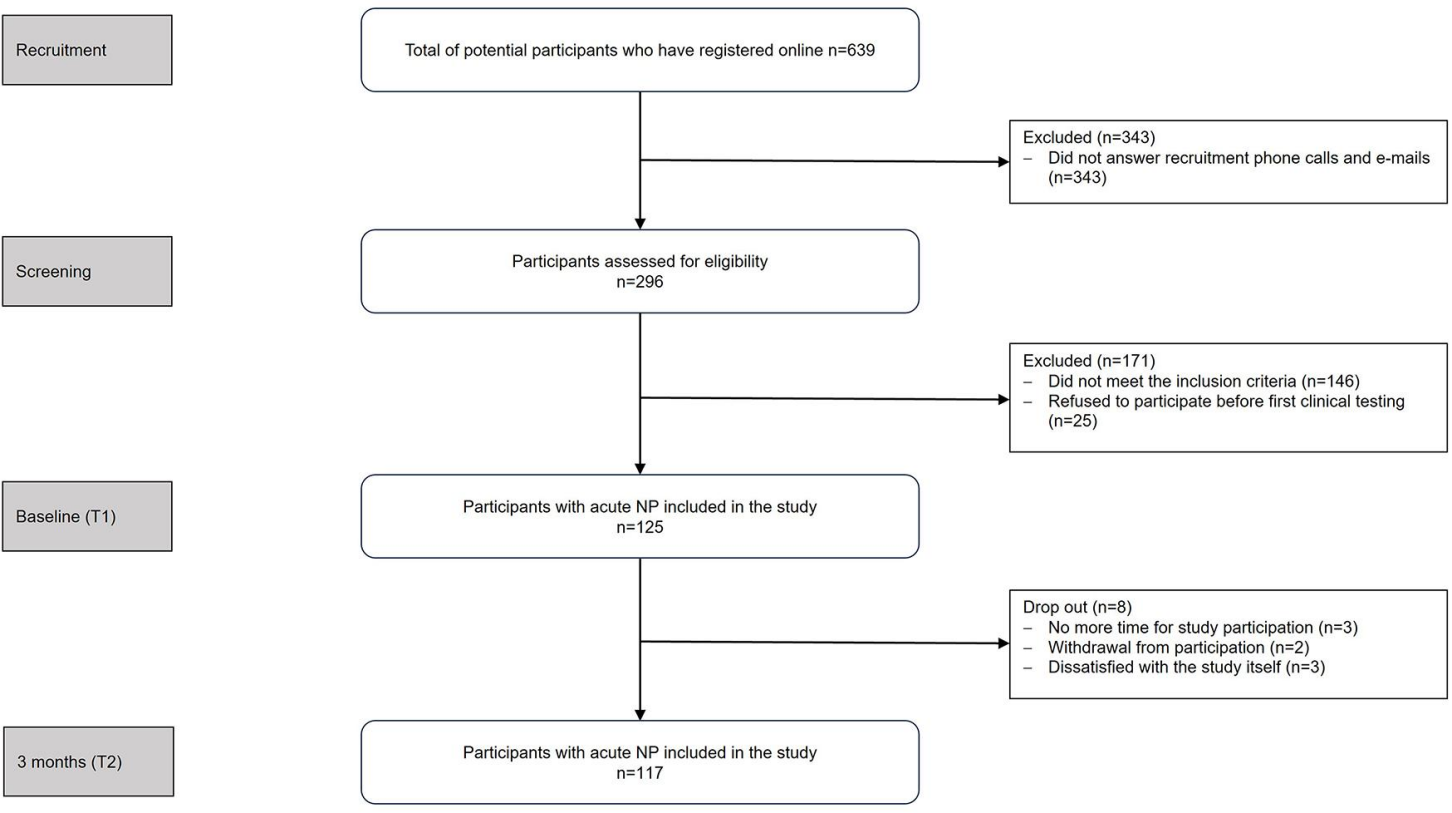
Study flowchart. N, number of participants; NP, neck pain, T1, time point 1 (within four weeks of pain onset); T2, time point 2 (three months after pain onset).

Participants were recruited from local physiotherapy practices, healthcare centers, universities, and companies. Interested individuals registered online and underwent an eligibility screening via survey and telephone interview. Of the 639 individuals who registered online, 514 were excluded prior to study enrollment due to non-response, failure to meet the inclusion criteria (as determined during telephone screening), or withdrawal. No data were collected from these individuals. Figure 1 shows the study flow chart. Before the first appointment (T1), detailed study information was provided, and informed consent was obtained.

### Data collection

Data were collected and managed using REDCap tools Version 15.5.3 hosted at the Zurich University of Applied Sciences (33, 34). Participants received a personal link to complete the online survey, and reminders were sent if it was not completed within three days. Participants were invited to a clinical testing center to collect hair samples.

### Measurements

#### Baseline characteristics

Sociodemographic data, including educational level, emp status, medication use, and medical consultations, were recorded. Furthermore, psychological variables were assessed to provide a broad overview of the participants' initial conditions, considering factors that might influence their experiences and behaviors throughout the study. For this purpose, the Depression Anxiety Stress Scale–21 (35), the Pain Vigilance and Awareness Questionnaire (36), and the State Anxiety Scale from the State-Trait Anxiety Inventory (37). Lifestyle factors, such as smoking status and sleep quality, were assessed. Additionally, physical activity levels were measured using the Physical Activity Questionnaire—Short Form (38).

#### Pain

Pain intensity was measured using the PainDETECT questionnaire (39). The first component of the questionnaire consists of seven questions regarding pain and pain quality, which demonstrates adequate internal consistency (Cronbach's alpha = 0.83) (39). Each item can be rated from 0 (no pain) to 10 (maximal pain) using the Numeric Rating Scale (NRS). The mean pain intensity is computed from the present, the strongest, and the average pain during the last four weeks, reflecting items 1–3. The minimal important change for the NRS is proposed as two points (40). The test-retest reliability and construct validity of the NRS scale are high, with coefficients of 0.96 and 0.95 (41).

#### Disability

Disability was assessed using the German version of the Neck Disability Index (NDI-G) (42). The self-administered questionnaire includes ten items referring to activities (personal care, lifting, driving, work, sleeping, concentration, reading, and recreation) and pain (pain intensity and headache). Each item is scored from 0 (no pain or functional limitation) to 5 points (worst pain and maximum limitation). The sum score ranges from a minimum of 0 points (no disability) to a maximum score of 50 points (total disability). This score is then converted into a percentage (0–100) (43). The minimal important change for the NDI-G is proposed as three points (40). With an ICC value of 0.92, NDI-G demonstrated high reliability. Cronbach's alpha is 0.96, indicating a high correlation among the items on the scale (42).

#### Activity patterns

The Avoidance Endurance Fast Screening tool was selected for its concise and time-efficient format, making it well-suited for clinical use (44). It was developed from the 37-item Avoidance-Endurance Questionnaire (45) by reducing the number of items and scales while maintaining internal validity (44). The Avoidance Endurance Fast Screening tool consists of nine items, with seven forming the Pain Persistence Scale and two constituting the Depressive Mood Scale. The Pain Persistence Scale demonstrates a sensitivity of 0.93 and a specificity of 0.81, with an AUC of 0.87 (95% CI: 0.80–0.93). The Depressive Mood Scale shows a sensitivity of 0.82 and a specificity of 0.92, with an AUC of 0.87 (95% CI: 0.80–0.94) (44).

Each item in the Pain Persistence Scale is rated on a Likert scale from 0 (never) to 6 (always) and is completed twice to capture behaviors related to mild and severe pain. The two items in the Depressive Mood Scale assess an individual's general mood over the previous two weeks. Response options of the first item include: “I can enjoy things just as much as I used to” (scored 0) or “I can no longer enjoy things as much as I used to” (scored 1). The second item examines decision-making difficulty, offering the responses: “I am as decisive as ever” (scored 0) or “I find it harder than usual to make decisions” (scored 1). Based on the established classification criteria (44), the screening questionnaire categorizes participants into one of four activity patterns: eustress persistence, distress persistence, activity pacing, or fear avoidance.

#### Stress

**Subjective measurement:** The Stress and Coping Inventory (SCI) evaluates an individual's current and long-term stress levels, as well as their physical and mental symptoms of stress (46). It consists of five scales: “Stress due to uncertainty”, “Stress due to excessive demands”, “Stress due to loss and negative events that have actually occurred”, “Physical and psychological stress symptoms” and “Dealing with stress (coping)”. This study used three of the five scales:

1. The “Stress due to uncertainty” scale consists of seven items that assess the degree to which participants felt burdened by specific uncertainties over the past three months. Respondents rate their experiences using a Likert scale ranging from 1 (not at all burdened) to 7 (very heavily burdened), resulting in a maximum number of 49 points. The items address key areas of life, including finances, housing, employment or training, relationships, health, and personal expectations. The scale demonstrated good internal consistency (*α* = .72) (46).
2. The “Stress due to excessive demands” scale comprises seven items that assess the extent to which participants felt overwhelmed by various events and problems over the past three months. Responses are rated on a Likert scale from 1 (not at all overwhelmed) to 7 (extremely overwhelmed), resulting in a maximum number of 49 points. The items address key areas of life, including finances, housing, employment or training, relationships, health, and personal expectations. The scale demonstrated good internal consistency (*α* = .69) (46).
3. The “Physical and psychological stress symptoms” scale comprises 13 items that assess physical and psychological symptoms associated with stress. Participants were asked about symptoms experienced in the past six months and rated them on a Likert scale from 1 (strongly disagree) to 4 (strongly agree), resulting in a maximum number of 52 points. The items cover a range of symptoms, including sleep disturbances, stomach pressure, headache, sadness, lack of motivation, significant weight changes, reduced sexual desire, withdrawal, concentration difficulties, and nightmares. The scale achieves an excellent reliability (*α* = .86) (46).

**Objective Measurement:** Hair Cortisol and Cortisone Concentration were used as objective biomarkers of long-term stress exposure. Hair Cortisol Concentration measures stress objectively with good test-retest reliability (*r* = .73) (47). It is robust against hair-related factors such as natural hair color (48). In addition, cortisone was included as a complementary marker to capture long-term hormone regulation better. It may enhance the validity of stress assessment by providing supplementary information and reflecting perceived stress across various populations (49–52).

The hair samples were collected at the posterior vertex as close to the head as possible and packaged for dispatch according to the specified protocol (53). To maintain sample integrity, they were stored in a sealed, dark container until analysis. For transportation, samples were shipped in multiple layers of protective packaging to prevent damage or contamination. Cortisol and cortisone concentrations were quantified in the laboratory of Prof. Kirschbaum using liquid chromatography–tandem mass spectrometry (LC-MS/MS), following the established protocol of this laboratory (53). The proximal 1 cm segment of hair was analyzed to assess hormone levels for the past month (Cortisol 1 month, Cortisone 1 month). The subsequent 3 cm segment was used to reflect cumulative secretion over the previous three months (Cortisol 3 months, Cortisone 3 months) (49). If hair length was insufficient, only the proximal segment was analyzed. Because men generally have higher hair cortisol levels, measured in pg/mg, than women, it is essential to interpret hair cortisol levels differently for men and women (48).

### Data analysis

Analyses were conducted using the statistical software R version 4.2.1–4.4.3; the complete R code is available in the Git repository (https://github.com/RitaMorf/Regression-3-months).

Data from three participants were missing for the analysis of activity patterns and the Stress and Coping Inventory at T2. Hair samples were not collected from six participants at T1 and nine at T2 due to lack of consent. To ensure accurate cortisol/cortisone results, nine outliers were excluded at T1 and ten were excluded at T2. Additionally, data on pain intensity and disability at T2 were missing for 18 participants. Outliers in hair cortisol and cortisone concentrations were defined as values exceeding the 95th percentile based on unpublished reference values from a larger cohort of 13,354 participants (mean age 48.1 ± 24.0 years, 69% female) (Clemens Kirschbaum, personal communication).

#### Sample characteristics

Descriptive statistics were used to summarize the baseline characteristics of the study sample, as presented in Table 1. Additionally, these analyses were used to screen for potential data outliers and assess the distribution of the collected variables.

**Table 1:** Baseline characteristics. Adapted from (26).

Variable	Mean (SD)	Range	95% CI
Sociodemographic variables			
Age (years)	29.4 (9.2)	18–65	(27.73, 30.98)
Female (%)	81 (66)		
Educational level			
Apprenticeship (%)	9 (7.3)		
Higher education: university (%)	104 (84.6)		
Other (%)	10 (8.1)		
Employment			
Unemployed (%)	3 (2.4)		
Part-time (%)	41 (33.3)		
Full-time (%)	31 (25.2)		
In training (%)	48 (39.0)		
Medication			
No medication (%)	32 (26.0)		
Painkillers (%)	20 (16.3)		
Opioids (%)	0		
Antidepressants (%)	2 (1.6)		
Muscle relaxants (%)	4 (3.3)		
Cannabis (%)	3 (2.4)		
Medical consultations			
No consultation of medical professionals (%)	51 (41.5)		
General practitioner (%)	24 (19.5)		
Therapy (physio, chiro, psycho, massage) (%)	41 (33.3)		
Neck pain-related variables			
Pain: PainDETECT, 0–10	4.93 (1.61)	1.33–8.33	(4.64, 5.21)
Disability: NDI 0–100%	22.35 (10.05)	4–50	(20.54, 24.17)
Psychological variables			
Depression-Scale: DASS21	7.93 (7.82)	0–40	(6.55, 9.32)
Stress-Scale: DASS21	13.62 (8.63)	0–38	(12.10, 15.15)
Anxiety: STAI-S	43.99 (9.88)	23–70	(42.25, 45.74)
Pain Vigilance: PVAQ	36.54 (9.98)	12–63	(34.78, 38.31)
Lifestyle Factors			
Physical Activity: IPAQ-SF (min/week)	4,135.7 (3,594.71)	297–19,836	(3,495.17, 4,776.17)
Sedentary activity: IPAQ-SF (min/week)	491.1 (252.51)	60–2,400	(446.44, 535.69)
Smoker (%)	10 (8.1)		
Sleep quality: 0–10	4.40 (2.08)	0–9	(4.04, 4.78)
Activity Patterns: AE-FS			
Pain Persistence Scale	3.23 (1.12)	1–6	(3.04, 3.43)
Depressive Mood Scale	0.91 (0.77)	0–2	(0.77, 1.05)
Subjective Stress: SCI			
“Stress due to uncertainty”	20.65 (8.16)	7–39	(19.23, 22.07)
“Stress due to excessive demands”	17.22 (6.57)	6–42	(16.08, 18.37)
“Physical and psychological stress symptoms”	12.54 (6.50)	1–27	(11.41, 13.67)
Objective Stress: Hair Cortisol/Cortisone Concentration			
Cortisol 1 month male (pg/mg)	3.18 (1.80)	1.21–10.1	(2.59, 3.77)
Cortisol 3 months male (pg/mg)	2.97 (1.28)	0.72–7.66	(2.51, 3.42)
Cortisol 1 month female (pg/mg)	3.37 (2.34)	0.29–14.0	(2.83, 3.91)
Cortisol 3 months female (pg/mg)	3.05 (2.48)	0.25–14.1	(2.45, 3.64)
Cortisone 1 month male (pg/mg)	13.5 (4.92)	5.85–29.9	(11.8, 15.2)
Cortisone 3 months male (pg/mg)	12.0 (4.76)	4.22–27.9	(10.3, 13.6)
Cortisone 1 month female (pg/mg)	13.6 (6.51)	0.23–29.4	(12.0, 15.1)
Cortisone 3 months female (pg/mg)	9.17 (5.21)	0.41–24.9	(7.92, 10.4)

Numbers are means (standard deviations) of participants unless stated otherwise.

95% CI, 95% confidence interval; AE-FS, avoidance-endurance fast screen; DASS21, depression anxiety stress scale; IPAQ-SF, international physical activity questionnaire short form; Mean (SD, standard deviation); NDI, neck disability index; PainDETECT, numeric rating scale (0–10); PVAQ, pain vigilance and awareness questionnaire; sleep quality: 0 = very bad, 10 = very good, SCI, stress and coping inventory; STAI-S, state-trait anxiety inventory-state.

#### Correlations of the subjective and objective stress measures

Pearson correlations were calculated to examine the between subjective stress measures—including the scales “Stress due to uncertainty”, “Stress due to excessive demands” and “Physical and psychological stress symptoms”—and objective stress measures (Cortisol 1 month/Cortisone 1 month and Cortisol 3 months/Cortisone 3 months) at T1.

#### Associations between explanatory variables at T1 and pain intensity at T2

To assess the associations between the independent variables/fixed effects (activity patterns, objective stress, subjective stress, and pain intensity) in the acute phase (T1) and the dependent variables (disability and pain intensity) at three months (T2), a linear mixed-effects model (LMM) was employed. Age and sex were considered potential confounders and were therefore included as independent variables, while depression was treated as a covariate and subjects were modeled as random slopes.

Two different models were fitted to the data:

1. Pain intensity, activity patterns, Cortisol 1, Cortisone 1, subjective stress (T1) as a prognostic factor, and pain intensity at time point (T2) as the dependent variable.
2. Pain intensity, activity patterns, Cortisol 1, Cortisone 1, subjective stress at time point (T1) as a prognostic factor, and disability at time point (T2) as the dependent variable.

First, multicollinearity was assessed. A high correlation coefficient (*r* = .75) was observed between the SCI subscales “Stress due to uncertainty” and “Stress due to excessive demands”. To reduce redundancy and improve model stability, the latter variable was excluded After this exclusion a reduced correlation (*r* = .57) could be found. Multicollinearity was further evaluated using variance inflation factors. Model assumptions were checked using residual and posterior predictive plots, which are provided in the Supplementary Material. Based on these diagnostic checks, a Bayesian multilevel model for a continuous outcome was applied for pain intensity, whereas a Bayesian zero-inflated multilevel model was used for NDI. Missing data were handled via median imputation, and model parameters for linear mixed models were estimated using restricted maximum likelihood. For the outcome disability (NDI-G), posterior predictive checks and inspection of distributions supported the use of a zero-inflated negative binomial model, which provided the best fit to the data. For the outcome pain intensity, a student-t regression model yielded the best model fit, as indicated by posterior checks and residual diagnostics. Additionally, model fit was evaluated using pseudo-R^2^ values for generalized linear mixed models, reporting both marginal and conditional R^2^ (54). To account for inherent uncertainty in the data and model parameters, Bayesian multilevel default priors in brms were used. This approach enables a coherent quantification and propagation of uncertainty throughout the modeling process (55). The fixed effects estimates for pain intensity and disability were interpreted using the minimal clinically important change threshold for subjects with NP (40, 56).

## Results

### Baseline characteristics

Between January 2023 and May 2024, 125 participants with acute NP (mean age 29.4 ± 9.2 years, 66% female) were enrolled. Table 1 outlines the sample characteristics, is adapted from our previous publication (26), showing that most participants had a university-level education, painkiller consumption was generally low, the disability levels showed minor symptoms (mean NDI = 22.35%), and pain intensity was moderate (mean NRS = 4.93). Furthermore, participants were highly physically active, with a mean IPAQ-SF score of 4,135.7 MET (Metabolic Equivalent of Task) -min/week. This value is substantially above the 3,000 MET-min/week threshold, categorizing the sample as “highly active” according to IPAQ-SF guidelines (57).

After inclusion, reasons for loss to follow up after 3 months included time restrictions (*n* = 3), withdrawal from participation (*n* = 2), and dissatisfaction with the study itself (*n* = 3).

### Correlation of the subjective and objective stress measures at T1

#### Cortisol 1 month

The Pearson product-moment correlation revealed weak correlations between Cortisol 1 month and “Stress due to uncertainty” (*r* = −0.07, *n* = 107, *p* = 0.446, CI = −0.262–0.118), “Stress due to excessive demands” (*r* = 0.02, *n* = 107, *p* = 0.861, CI = −0.174–0.207) or “Physical and psychological stress symptoms” (*r* = −0.011, *n* = 107, *p* = 0.913, CI = −0.201–0.180). Scatterplots illustrating the associations between subjective and objective stress measures are provided in the Supplementary Material.

#### Cortisone 1 month

Weak correlations were found between Cortisone 1 month and “Stress due to uncertainty” (*r* = −0.01, *n* = 107, *p* = 0.913, 95% CI = −0.201–0.180), “Stress due to excessive demands” (*r* = 0.09, *n* = 107, *p* = 0.370, 95% CI = −0.104–0.274) or “Physical and psychological stress symptoms” (*r* = 0.04, *n* = 107, *p* = 0.667, 95% CI = −0.150–0.231).

#### Cortisol 3 months

The Pearson product-moment correlation revealed weak correlations between Cortisol 3 months and “Stress due to uncertainty” (*r* = 0.06, *n* = 102, *p* = 0.545, 95% CI = −0.135–0.252), “Stress due to excessive demands” (*r* = 0.23, *n* = 102, *p* = 0.021, 95% CI = 0.035–0.404) or “Physical and psychological stress symptoms” (*r* = 0.12, *n* = 102, *p* = 0.215, 95% CI = −0.072–0.311).

#### Cortisone 3 months

Weak correlations were found between Cortisone 3 months and “Stress due to uncertainty” (*r* = −0.07, *n* = 103, *p* = 0.479, 95% CI = −0.260–0.125), “Stress due to excessive demands” (*r* = 0.01, *n* = 103, *p* = 0.912, 95% CI = −0.183–0.204) or “Physical and psychological stress symptoms” (*r* = −0.06, *n* = 103, *p* = 0.542, 95% CI = −0.251–0.134).

### Associations between explanatory variables at T1 and pain intensity at T2

Pain intensity in acute NP (T1) [estimate: 0.871, 95% Credible Interval (CRI): 0.442–1.276] was associated with higher pain intensities after 3 months (T2). The activity patterns, subjective stress (“Stress due to uncertainty” and “Physical and psychological stress symptoms”), Cortisol 1, and Cortisone 1 demonstrated weak associations with pain intensity (T2). The model explained 59% of the variance, with an R^2^ of 0.592 (95% CRI: 0.295–0.937). Table 2 presents the fixed effects from the LMM, which evaluates the associations between the explanatory variables and pain intensity over a three-month period.

**Table 2:** Fixed effects estimates for pain intensity at T2.

			95% CI	
Explanatory Variables T1	Estimate	SE	2.5%	97.5%
Intercept	3.275	0.526	2.221	4.276
Age	−0.135	0.225	−0.573	0.297
Gender: female	0.201	0.483	−0.767	1.158
Depression	−0.202	0.291	−0.770	0.361
Cortisol	−0.026	0.260	−0.481	0.548
Cortisone	0.125	0.268	−0.395	0.674
Fear Avoidance	−0.406	0.898	−2.150	1.409
Distress Persistence	0.540	0.741	−0.920	1.976
Eustress Persistence	0.623	0.487	−0.379	1.576
Pain intensity	0.871	0.210	0.442	1.276
“Stress due to uncertainty”	0.374	0.256	−0.136	0.863
“Physical and psychological stress symptoms”	0.169	0.292	−0.389	0.760

CI, confidence interval; Cortisol, hair cortisol concentration; SE, standard error; Tl, time point 1 (within four weeks of pain onset). T2, time point 2 (three months after pain onset).

Activity Patterns: Activity Pacing was used as a reference.

Depression: measured with Depression Anxiety Stress Scale—21 (DASS-21), Pain intensity measured with PainDETECT questionnaire, “Stress due to uncertainty” and “Physical and psychological stress symptoms” measured with Stress and Coping Inventory (SCI).

### Associations between explanatory variables at T1 and disability at T2

Pain intensity in the acute phase (T1) (estimate: 0.171, 95% CRI: 0.011–0.309) and subjective stress (“Stress due to uncertainty”) (estimate: 0.210, 95% CRI: 0.048–0.381) were associated with higher disability after 3 months (T2). The activity patterns, “Physical and psychological stress symptoms”, Cortisol 1, and Cortisone 1 demonstrated weak associations with disability. The model explained 52% of the variance, with an R^2^ of 0.522 (95% CRI: 0.286–0.785). Table 3 presents the fixed effects from the LMM, which evaluates the associations between explanatory variables and disability over a 3-month period.

**Table 3:** Fixed effects estimates for disability at T2.

			95% CI	
Explanatory Variables T1	Estimate	SE	2.5%	97.5%
Intercept	2.580	0.190	2.252	2.953
Age	0.027	0.083	−0.114	0.186
Gender	0.197	0.169	−0.149	0.476
Depression	0.066	0.100	−0.114	0.272
Cortisol	0.122	0.081	−0.040	0.279
Cortisone	−0.152	0.095	−0.329	0.038
Fear Avoidance	0.058	0.292	−0.550	0.589
Distress Persistence	−0.169	0.222	−0.617	0.298
Eustress Persistence	0.228	0.158	−0.097	0.545
Pain intensity	0.171	0.080	0.011	0.309
“Stress due to uncertainty”	0.210	0.087	0.048	0.381
“Physical and psychological stress symptoms”	0.048	0.115	−0.149	0.277

CI, confidence interval; Cortisol, hair cortisol concentration; SE, standard error; T1, time point 1 within four weeks of pain onset); T2, time point 2 (three months after pain onset).

Activity Patterns: Activity Pacing was used as a reference.

Depression: measured with Depression Anxiety Stress Scale—21 (DASS-21), Pain intensity measured with PainDETECT questionnaire, “Stress due to uncertainty” and “Physical and psychological stress symptoms” measured with Stress and Coping Inventory (SCI).

## Discussion

The study investigated whether activity patterns and subjective and objective stress measurements, in the acute phase of NP, could serve as prognostic factors for the persistence of pain and disability. Furthermore, the study explored the correlation between subjective and objective assessments.

### Correlation of the subjective and objective stress measures at T1

The findings revealed weak correlations between objective and subjective stress measures, which adds to the existing body of inconsistent research (12, 13, 58). The subjective measurement method relied on an individual's perception of stress, which varies from person to person in terms of how situations are assessed, how stress is experienced, and its impact on the individual (59). In contrast, objective measures assess hair cortisol levels and provide quantifiable data that is independent of personal bias. While subjective measures offer insight into perceived stress, objective measures reveal the body's biological responses, highlighting the complex interplay between the psychological and physiological dimensions of stress (58). The two measurement methods also differed in terms of their timeframe. The SCI assessed perceived stress over the past three to six months. In contrast, hair cortisol concentrations reflected physiological stress over either the past month (Cortisol 1 month) or the past three months (Cortisol 3 months), encompassing the acute pain phase and beyond. To account for this difference, we calculated correlations for both cortisol assessments, which suggests that the lack of association may not be explained solely by timing differences. In a previously published analysis from the same dataset, we reported that Hair Cortisol values were notably low, raising the possibility that no relevant physiological stress response occurred (26). However, hair cortisol concentration can be affected by several confounding factors. Frequent hair washing and the use of oral contraceptives have been shown to correlate negatively with hair cortisol (58), while UV exposure may also reduce cortisol levels (60). In contrast, endurance athletes often exhibit higher cortisol concentrations in their hair (61). Such factors could have contributed to the variability observed in our sample.

Taken together, this suggests that although participants reported subjective stress, it may not have been strong or sustained enough to activate a biological response detectable through hair cortisol. These findings reinforce the idea that subjective and objective stress measures may not assess the same underlying construct and should be interpreted as complementary rather than interchangeable. Further research is needed to clarify the conditions under which psychological stress translates into physiological activation and how this dynamic influences pain persistence.

### Associations between explanatory variables and pain intensity and disability at T2

#### Stress and pain

Pain intensity at T1 was associated with both disability and pain intensity at 3-month follow-up. Objective and subjective stress measures were weakly associated with pain intensity at the 3-month follow-up. The only exception was a weak association between “Stress due to uncertainty” and disability; however, effect sizes were minimal and their magnitude falls below the minimal clinically important change threshold, and thus they are not considered clinically meaningful (40, 56). These findings partially align with previous findings linking psychological stress and disability in chronic NP (8). Given the low cortisol concentrations observed in our sample, it is possible that the reported subjective stress did not translate into a corresponding physiological stress reaction. This points to the possibility that contextual factors, such as life circumstances, coping resources, or the acute nature of symptoms, may play a more decisive role. However, it remains possible that stress becomes more influential as symptoms persist and functional limitations increase. Therefore, follow-up measurements at later time points may offer more relevant insights into the relationship between stress and persistent pain or disability.

Furthermore, the interaction between pain and stress has often been treated unidirectional, typically with stress viewed as a cause of pain (62). Whether stress acts as a prognostic factor or emerges as a consequence of persistent pain remains a complex question, as each can affect and reinforce the other (63). It is also conceivable that stress does not exclusively exacerbate pain but may, under certain circumstances, activate compensatory biochemical mechanisms with analgesic effects, such as the endogenous opioid system (64). These mechanisms may be particularly relevant in physically active individuals and could partly account for the absence of associations observed in the present study.

Further research is needed to better understand the complex and reciprocal relationship between stress and pain, particularly during the transition from acute to chronic phases.

#### Activity patterns

The present cohort showed weak associations between the different activity patterns and pain intensity or disability after three months. This is in line with earlier literature suggesting that fear avoidance or persistence are of limited importance during the acute or very early subacute phase, becoming more relevant only as pain persists (65, 66). Our recent findings in individuals with acute low back pain suggest that baseline eustress persistence, rather than fear avoidance, was associated with the development of persistent pain over one year (25). It is therefore conceivable that the three-month time point may represent a more meaningful starting point for prognostic long-term outcomes. Symptoms persisting beyond this period may be perceived as burdensome, which could increase their prognostic value for persistent pain and disability.

### Strengths and limitations

Objective and subjective stress are rarely assessed simultaneously, yet their combined evaluation offers meaningful insights by capturing both the individual's perceived stress and the physiological response of the body. In our study, however, the objective stress measurement via hair cortisol was limited by partial participant compliance, as not all agreed to provide hair samples, resulting in missing data and reduced statistical power of the cortisol analyses. Moreover, our sample was on average younger and predominantly female, both factors known to be associated with lower hair cortisol levels (48), which may have further reduced the variance necessary to detect correlations. These sample characteristics, together with the incomplete data, should be considered when interpreting the results.

This sample was not specifically recruited to address the present research question but represents a subset of participants from a larger project, which may limit the precision with which the findings address the specific aims of this study.

The recruitment via physiotherapy clinics proved difficult, as individuals with acute neck pain often delay seeking physiotherapeutic care. As an alternative, a large portion of the sample was recruited through mass email distributions at academic institutions. This approach resulted in a sample characterized by high educational attainment. This factor limits the generalizability of the findings.

Given that only a few consistent prognostic factors for the development of persistent pain and disability have been confirmed so far, this raises the question of whether current measurement tools truly capture the most relevant aspects. Other, less frequently assessed aspects of patients' experiences may play a decisive role in the transition from acute to chronic symptoms.

## Conclusion

This study found limited prognostic value of subjective and objective stress, as well as activity patterns, with pain and disability three months after acute neck pain onset. The lack of clinically relevant associations raises the question of whether we capture the most relevant aspects. Future research should explore whether other, less frequently assessed psychosocial or contextual aspects better reflect the transition to persistent pain and disability and consider extended follow-up time points.

## Data Availability

The raw data supporting the conclusions of this article will be made available by the authors, without undue reservation.

## References

[1] Fejer R Kyvik KO Hartvigsen J. The prevalence of neck pain in the world population: a systematic critical review of the literature. Eur Spine J. (2006) 15:834–48. 10.1007/s00586-004-0864-4 15999284 PMC3489448

[2] Hush JM Lin CC Michaleff ZA Verhagen A Refshauge KM. Prognosis of acute idiopathic neck pain is poor: a systematic review and meta-analysis. Arch Phys Med Rehabil. (2011) 92:824–9. 10.1016/j.apmr.2010.12.025 21458776

[3] Haldeman S Carroll L Cassidy JD Schubert J Nygren Å. The bone and joint decade 2000–2010 task force on neck pain and its associated disorders. J Manipulative Physiol Ther. (2009) 32:S7–9. 10.1016/j.jmpt.2008.11.005 19251077

[4] Aldabbas MM Tanwar T Iram I Ghrouz A Veqar Z. Predictors of persistent pain in patients with acute neck pain treated with physical therapy: a prospective study with 2 years follow up. Musculoskelet Care. (2023) 21(4):980–6. 10.1002/msc.1775 37139892

[5] Carroll LJ Hogg-Johnson S Van Der Velde G Haldeman S Holm LW Carragee EJ Course and prognostic factors for neck pain in the general population. J Manipulative Physiol Ther. (2009) 32:S87–96. 10.1016/j.jmpt.2008.11.013 19251079

[6] Wirth B Humphreys BK Peterson C. Importance of psychological factors for the recovery from a first episode of acute non-specific neck pain—a longitudinal observational study. Chiropr Man Ther. (2016) 24:9. 10.1186/s12998-016-0090-2 PMC4793758 26985362

[7] McEwen BS. Physiology and neurobiology of stress and adaptation: central role of the brain. Physiol Rev. (2007) 87:873–904. 10.1152/physrev.00041.2006 17615391

[8] Ortego G Villafañe JH Doménech-García V Berjano P Bertozzi L Herrero P. Is there a relationship between psychological stress or anxiety and chronic nonspecific neck-arm pain in adults? A systematic review and meta-analysis. J Psychosom Res. (2016) 90:70–81. 10.1016/j.jpsychores.2016.09.006 27772562

[9] Grotle M Brox JI Glomsrød B Lønn JH Vøllestad NK. Prognostic factors in first-time care seekers due to acute low back pain. Eur J Pain. (2007) 11:290–8. 10.1016/j.ejpain.2006.03.004 16677837

[10] Tramel W Schram B Canetti E Orr R. An examination of subjective and objective measures of stress in tactical populations: a scoping review. Healthcare. (2023) 11:2515. 10.3390/healthcare11182515 37761712 PMC10530665

[11] Staufenbiel SM Penninx BWJH Spijker AT Elzinga BM Van Rossum EFC. Hair cortisol, stress exposure, and mental health in humans: a systematic review. Psychoneuroendocrinology. (2013) 38:1220–35. 10.1016/j.psyneuen.2012.11.015 23253896

[12] Gidlow CJ Randall J Gillman J Silk S Jones MV. Hair cortisol and self-reported stress in healthy, working adults. Psychoneuroendocrinology. (2016) 63:163–9. 10.1016/j.psyneuen.2015.09.022 26447679

[13] Weckesser LJ Dietz F Schmidt K Grass J Kirschbaum C Miller R. The psychometric properties and temporal dynamics of subjective stress, retrospectively assessed by different informants and questionnaires, and hair cortisol concentrations. Sci Rep. (2019) 9:1098. 10.1038/s41598-018-37526-2 30705360 PMC6355861

[14] Van Damme S Kindermans H. A self-regulation perspective on avoidance and persistence behavior in chronic pain: new theories, new challenges? Clin J Pain. (2015) 31:115–22. 10.1097/AJP.0000000000000096 24662496

[15] Hasenbring MI Andrews NE Ebenbichler G. Overactivity in chronic pain, the role of pain-related endurance and neuromuscular activity: an interdisciplinary, narrative review. Clin J Pain. (2020) 36:162–71. 10.1097/AJP.0000000000000785 31833914

[16] Hasenbring MI Verbunt JA. Fear-avoidance and endurance-related responses to pain: new models of behavior and their consequences for clinical practice. Clin J Pain. (2010) 26:747–53. 10.1097/AJP.0b013e3181e104f2 20664333

[17] Kindermans HPJ Roelofs J Goossens MEJB Huijnen IPJ Verbunt JA Vlaeyen JWS. Activity patterns in chronic pain: underlying dimensions and associations with disability and depressed mood. J Pain. (2011) 12:1049–58. 10.1016/j.jpain.2011.04.009 21704568

[18] Vlaeyen JWS Crombez G Linton SJ. The fear-avoidance model of pain. Pain. (2016) 157:1588–9. 10.1097/j.pain.0000000000000574 27428892

[19] Smith J Russo LM Santayana N. Fear avoidance predicts persistent pain in young adults with low back pain: a prospective study. J Orthop Sports Phys Ther. (2021) 51(8):383–91. 10.2519/jospt.2021.9828 33998262 PMC8328870

[20] Slepian P Ankawi B France C. Longitudinal analysis supports a fear-avoidance model that incorporates pain resilience alongside pain catastrophizing. Ann Behav Med Publ Soc Behav Med. (2019) 54(5):335–45. 10.1093/abm/kaz051 31711106

[21] Kachur S. Understanding and treating fear of pain. Physiother Can. (2008) 60:196–7. 10.3138/physio.60.2.196

[22] Gatchel RJ Neblett R Kishino N Ray CT. Fear-avoidance beliefs and chronic pain. J Orthop Sports Phys Ther. (2016) 46:38–43. 10.2519/jospt.2016.0601 26828236

[23] Zale EL Ditre JW. Pain-related fear, disability, and the fear-avoidance model of chronic pain. Curr Opin Psychol. (2015) 5:24–30. 10.1016/j.copsyc.2015.03.014 25844393 PMC4383173

[24] Kroska EB. A meta-analysis of fear-avoidance and pain intensity: the paradox of chronic pain. Scand J Pain. (2016) 13:43–58. 10.1016/j.sjpain.2016.06.011 28850534

[25] Hotz-Boendermaker S Surbeck U Morf R Pfeiffer F. Persistence, not avoidance, is associated with low back pain—an observational cohort study. Eur J Pain. (2024) 29(2):e4728. 10.1002/ejp.4728 39350320 PMC11671328

[26] Morf R Leah R Jürgen D Monika H Anna E Sabina H-B. Frequencies of persistence, activity pacing, fear avoidance and general stress in acute neck pain. Compr Psychoneuroendocrinology. (2025) 23:100308. 10.1016/j.cpnec.2025.100308 PMC12221366 40606649

[27] Sudhaus S Fricke B Stachon A Schneider S Klein H von Düring M Salivary cortisol and psychological mechanisms in patients with acute versus chronic low back pain. Psychoneuroendocrinology. (2009) 34:513–22. 10.1016/j.psyneuen.2008.10.011 19028020

[28] Van Uum SHM Sauvé B Fraser LA Morley-Forster P Paul TL Koren G. Elevated content of cortisol in hair of patients with severe chronic pain: a novel biomarker for stress: short communication. Stress. (2008) 11:483–8. 10.1080/10253890801887388 18609301

[29] Cuschieri S. The STROBE guidelines. Saudi J Anaesth. (2019) 13:31. 10.4103/sja.SJA_543_18 PMC6398292 30930717

[30] Morf R Reicherzer L Degenfellner J Ernst MJ Pfeiffer F Luomajoki H Study protocol: activity patterns and stress—prognostic factors for pain persistence and disability in acute neck pain. A one year prospective inception cohort study. OSF. (2024). 10.17605/OSF.IO/DFY2J

[31] Ashina S Bendtsen L Lyngberg AC Lipton RB Hajiyeva N Jensen R. Prevalence of neck pain in migraine and tension-type headache: a population study. Cephalalgia. (2015) 35:211–9. 10.1177/0333102414535110 24853166

[32] Liang Z Galea O Thomas L Jull G Treleaven J. Cervical musculoskeletal impairments in migraine and tension type headache: a systematic review and meta-analysis. Musculoskelet Sci Pract. (2019) 42:67–83. 10.1016/j.msksp.2019.04.007 31054485

[33] Harris PA Taylor R Thielke R Payne J Gonzalez N Conde JG. Research electronic data capture (REDCap)—a metadata-driven methodology and workflow process for providing translational research informatics support. J Biomed Inform. (2009) 42:377–81. 10.1016/j.jbi.2008.08.010 18929686 PMC2700030

[34] Harris PA Taylor R Minor BL Elliott V Fernandez M O’Neal L The REDCap consortium: building an international community of software platform partners. J Biomed Inform. (2019) 95:103208. 10.1016/j.jbi.2019.103208 31078660 PMC7254481

[35] Nilges P Essau C. Die depressions-angst-stress-skalen: der DASS—ein screeningverfahren nicht nur für schmerzpatienten. Schmerz. (2015) 29:649–57. 10.1007/s00482-015-0019-z 26205682

[36] Kunz M Capito ES Horn-Hofmann C Baum C Scheel J Karmann AJ Psychometric properties of the German version of the pain vigilance and awareness questionnaire (PVAQ) in pain-free samples and samples with acute and chronic pain. Int J Behav Med. (2017) 24:260–71. 10.1007/s12529-016-9585-4 27481106 PMC5344944

[37] Spielberger CD. State-trait anxiety inventory for adults. APA PsycTest. (2012. 10.1037/t06496-000

[38] Lee PH Macfarlane DJ Lam T Stewart SM. Validity of the international physical activity questionnaire short form (IPAQ-SF): a systematic review. Int J Behav Nutr Phys Act. (2011) 8:115. 10.1186/1479-5868-8-115 22018588 PMC3214824

[39] Freynhagen R Baron R Gockel U Tölle TR. Pain *DETECT* : a new screening questionnaire to identify neuropathic components in patients with back pain. Curr Med Res Opin. (2006) 22:1911–20. 10.1185/030079906X132488 17022849

[40] Ostelo RWJG Deyo RA Stratford P Waddell G Croft P Von Korff M Interpreting change scores for pain and functional status in low back pain: towards international consensus regarding minimal important change. Spine. (2008) 33:90–4. 10.1097/BRS.0b013e31815e3a10 18165753

[41] Hawker GA Mian S Kendzerska T French M. Measures of adult pain: visual analog scale for pain (VAS pain), numeric rating scale for pain (NRS pain), McGill pain questionnaire (MPQ), short-form McGill pain questionnaire (SF-MPQ), chronic pain grade scale (CPGS), short form-36 bodily pain scale (SF). Arthritis Care Res. (2011) 63:S240–52. 10.1002/acr.20543 22588748

[42] Swanenburg J Humphreys K Langenfeld A Brunner F Wirth B. Validity and reliability of a German version of the neck disability index (NDI-G). Man Ther. (2014) 19:52–8. 10.1016/j.math.2013.07.004 23920153

[43] Vernon H Mior S. The neck disability index: a study of reliability and validity. J Manipulative Physiol Ther. (1991) 14:409–15. PMID: 1834753

[44] Wolff SV Willburger R Hallner D Rusu AC Rusche H Schulte T Avoidance-endurance fast-screen (AE-FS): inhalts- und vorhersagevalidität eines 9-item-screeninginstruments für patienten mit unspezifischen subakuten rückenschmerzen. Schmerz. (2018) 32:283–92. 10.1007/s00482-018-0310-x 29987513

[45] Hasenbring MI Hallner D Rusu AC. Fear-avoidance- and endurance-related responses to pain: development and validation of the avoidance-endurance questionnaire (AEQ). Eur J Pain. (2009) 13:620–8. 10.1016/j.ejpain.2008.11.001 19101182

[46] Satow L. SCI—stress- und coping-inventar. Leibniz-Institut für Psychologie. (2012. 10.23668/PSYCHARCHIVES.4604

[47] Stalder T Steudte S Miller R Skoluda N Dettenborn L Kirschbaum C. Intraindividual stability of hair cortisol concentrations. Psychoneuroendocrinology. (2012) 37:602–10. 10.1016/j.psyneuen.2011.08.007 21917384

[48] Dettenborn L Tietze A Kirschbaum C Stalder T. The assessment of cortisol in human hair: associations with sociodemographic variables and potential confounders. Stress. (2012) 15:578–88. 10.3109/10253890.2012.654479 22356099

[49] Stalder T Kirschbaum C. Analysis of cortisol in hair—state of the art and future directions. Brain Behav Immun. (2012) 26:1019–29. 10.1016/j.bbi.2012.02.002 22366690

[50] Scharlau F Pietzner D Vogel M Gaudl A Ceglarek U Thiery J Evaluation of hair cortisol and cortisone change during pregnancy and the association with self-reported depression, somatization, and stress symptoms. Stress Amst Neth. (2018) 21:43–50. 10.1080/10253890.2017.1392507 29073819

[51] Feeney JC O’Halloran AM Kenny RA. The association between hair cortisol, hair cortisone, and cognitive function in a population-based cohort of older adults: results from the Irish longitudinal study on ageing. J Gerontol Ser A. (2018) 75(2):257–65. 10.1093/gerona/gly258 30412218

[52] Staufenbiel SM Penninx BWJH de Rijke YB van den Akker ELT van Rossum EFC. Determinants of hair cortisol and hair cortisone concentrations in adults. Psychoneuroendocrinology. (2015) 60:182–94. 10.1016/j.psyneuen.2015.06.011 26176863

[53] Kirschbaum C Tietze A Skoluda N Dettenborn L. Hair as a retrospective calendar of cortisol production—increased cortisol incorporation into hair in the third trimester of pregnancy. Psychoneuroendocrinology. (2009) 34:32–7. 10.1016/j.psyneuen.2008.08.024 18947933

[54] Nakagawa S Schielzeth H. A general and simple method for obtaining R2 from generalized linear mixed-effects models. Methods Ecol Evol. (2013) 4:133–42. 10.1111/j.2041-210x.2012.00261.x

[55] Bürkner P-C. Advanced bayesian multilevel modeling with the R package brms. arXiv Preprint ArXiv:1705. (2017):11123. 10.48550/arXiv.1705.11123

[56] Pool JJM Ostelo RWJG Hoving JL Bouter LM De Vet HCW. Minimal clinically important change of the neck disability index and the numerical rating scale for patients with neck pain. Spine. (2007) 32:3047–51. 10.1097/BRS.0b013e31815cf75b 18091500

[57] Craig CL Marshall AL Sjöström MM Bauman AE Booth ML Ainsworth BE International physical activity questionnaire: 12-country reliability and validity. Med Sci Sports Exerc. (2003) 35:1381–95. 10.1249/01.MSS.0000078924.61453.FB 12900694

[58] Stalder T Steudte-Schmiedgen S Alexander N Klucken T Vater A Wichmann S Stress-related and basic determinants of hair cortisol in humans: a meta-analysis. Psychoneuroendocrinology. (2017) 77:261–74. 10.1016/j.psyneuen.2016.12.017 28135674

[59] Schwarzer R. Streß, Angst und Handlungsregulation. 4., überarb. Aufl Stuttgart Berlin Köln: Kohlhammer (2000). p. 274.

[60] Wester VL van der Wulp NRP Koper JW de Rijke YB van Rossum EFC. Hair cortisol and cortisone are decreased by natural sunlight. Psychoneuroendocrinology. (2016) 72:94–6. 10.1016/j.psyneuen.2016.06.016 27392216

[61] Skoluda N Dettenborn L Stalder T Kirschbaum C. Elevated hair cortisol concentrations in endurance athletes. Psychoneuroendocrinology. (2012) 37:611–7. 10.1016/j.psyneuen.2011.09.001 21944954

[62] Linton SJ. A review of psychological risk factors in back and neck pain. Spine. (2000) 25:1148–56. 10.1097/00007632-200005010-00017 10788861

[63] Hannibal KE Bishop MD. Chronic stress, cortisol dysfunction, and pain: a psychoneuroendocrine rationale for stress management in pain rehabilitation. Phys Ther. (2014) 94:1816–25. 10.2522/ptj.20130597 25035267 PMC4263906

[64] Pilozzi A Carro C Huang X. Roles of β-endorphin in stress, behavior, neuroinflammation, and brain energy metabolism. Int J Mol Sci. (2021) 22:338. 10.3390/ijms22010338 PMC7796446 33396962

[65] Hasenbring M Hallner D Klasen B. Psychological mechanisms in the transition from acute to chronic pain: over- or underrated? Schmerz. (2001) 15:442–7. 10.1007/s004820100030 11793149

[66] Hasenbring M. Attentional control of pain and the process of chronification. Prog Brain Res. (2000) 129:525–34. 10.1016/S0079-6123(00)29038-9 11098715

