# Supplementary material for: Prognostic value of activity patterns and stress measures for persistent pain and disability in acute neck pain: a 3-month follow-up study
Source: Front Pain Res (Lausanne). 2025 Nov 13;6:1686389. doi: 10.3389/fpain.2025.1686389 (PMC12657464; doi:10.3389/fpain.2025.1686389)

**Supplementary File**

**Model checks for Linear Mixed-Effects Model**

Figure 1 Posterior predictive check for pain intensity with a Bayesian multilevel model for a continuous outcome


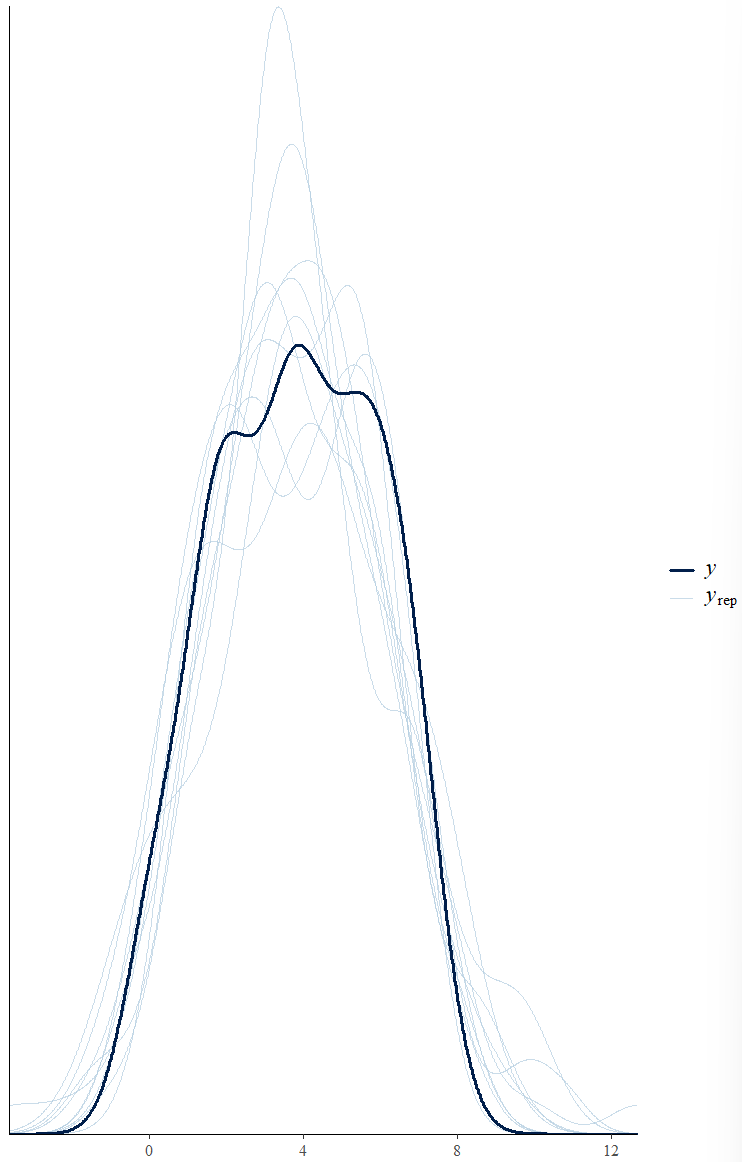


Figure 2 Posterior predictive check for NDI with a Bayesian zero-inflated multilevel model for a continuous outcome


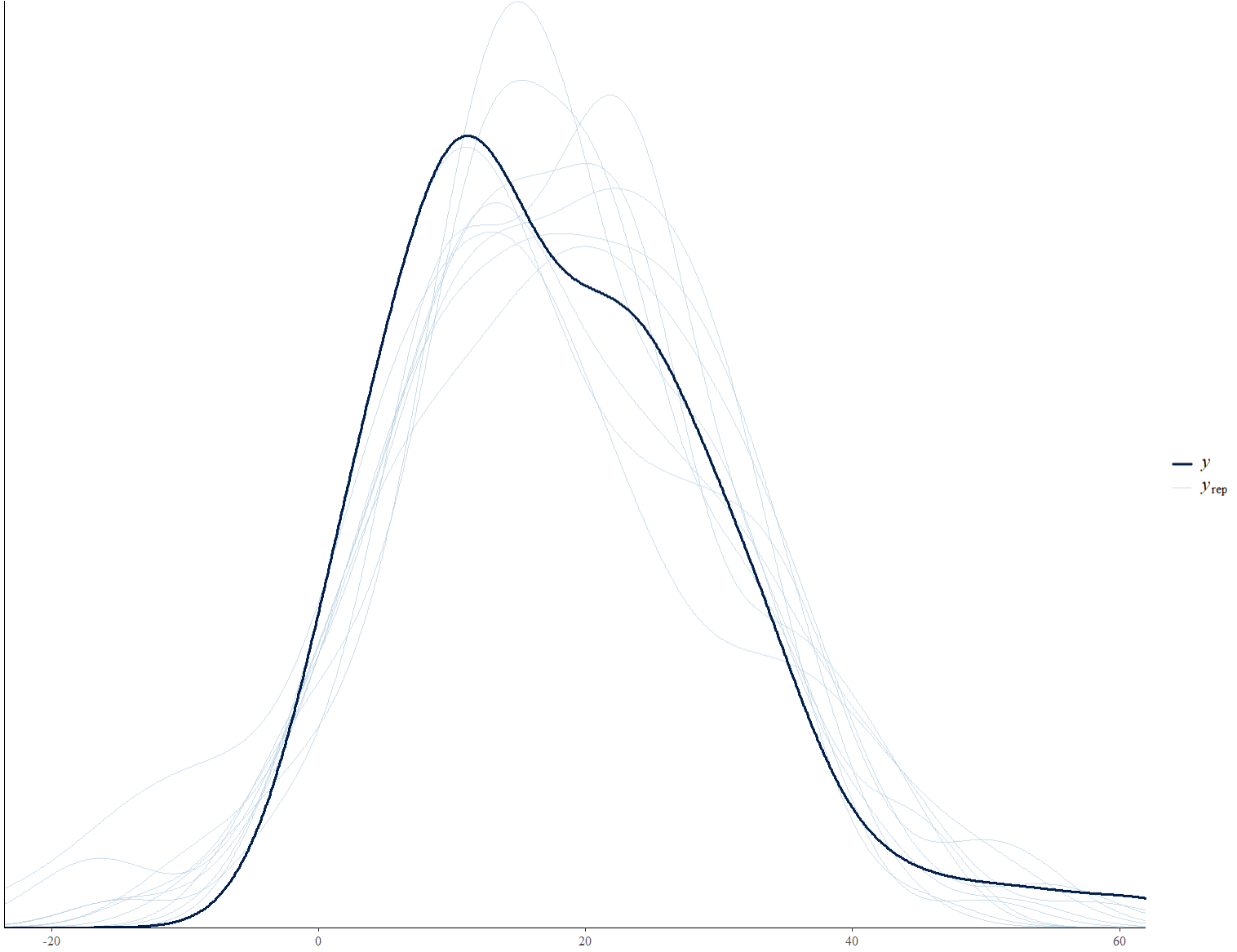

Supplement: Supplementary file 1 [file Datasheet1.docx]
